# *In vivo* imaging of bacterial colonization of the lower respiratory tract in a baboon model of *Bordetella pertussis* infection and transmission

**DOI:** 10.1038/s41598-018-30896-7

**Published:** 2018-08-16

**Authors:** Thibaut Naninck, Loïc Coutte, Céline Mayet, Vanessa Contreras, Camille Locht, Roger Le Grand, Catherine Chapon

**Affiliations:** 1 CEA – Université Paris Sud 11 – INSERM U1184, Immunology of Viral Infections and Autoimmune Diseases, IDMIT Department, IBFJ, Fontenay-aux-Roses & Le Kremlin-Bicêtre, France; 2 Université de Lille, CNRS UMR8204, INSERM U1019, Center for Infection and Immunity of Lille, Institut Pasteur de Lille, Lille, France

## Abstract

Recent whooping cough (pertussis) outbreaks in many countries highlight the crucial need for a better understanding of the pathogenesis of *Bordetella pertussis* infection of the respiratory tract. The baboon is a recently described preclinical model for the study of *B. pertussis* infection and may be ideal for the evaluation of new pertussis vaccines. However, many pathophysiological aspects, including bacterial localization and interactions, have yet to be described in this model. Here, we used a baboon model of infection with a fluorescent GFP-expressing *B. pertussis* strain, derived from European clinical isolate B1917. Juvenile baboons were used to evaluate susceptibility to infection and transmission. Non-invasive *in vivo* imaging procedures, using probe-based confocal endomicroscopy coupled with bronchoscopy, were developed to track fluorescent bacterial localization and cellular interactions with host cells in the lower respiratory tract of infected animals. All B1917-GFP-challenged animals developed classical pertussis symptoms, including paroxysmal cough, nasopharyngeal colonization, and leukocytosis. *In vivo* co-localization with antigen presenting cells and progressive bacterial colonization of the lower airways were also assessed by imaging during the first weeks of infection. Our results demonstrate that *in vivo* imaging can be used to assess bacterial colonization and to point out interactions in a baboon model of pertussis.

## Introduction

Whooping cough, or pertussis, is mainly caused by *Bordetella pertussis* infection of the airways. There were 24.1 million estimated pertussis cases in young children and 160,700 associated deaths world-wide in 2014^1^. The main symptoms observed are a prolonged characteristic paroxysmal cough, sometimes coupled with mild fever and leukocytosis. This disease affects mostly children and may be life-threatening in infants, in whom hyper-leukocytosis may lead to pulmonary hypertension and respiratory failure. High vaccine coverage globally with diphtheria-tetanus-pertussis vaccines^2^ (86% in 2016) has failed to control the disease, which is now re-emerging in several developed countries, including the USA, the UK, and Australia^3^. The reasons for the resurgence of pertussis are still a matter of debate and may include better detection due to enhanced surveillance and diagnostic tools, strain adaptation to escape vaccine-induced immunity, waning immunity, and asymptomatic carriage and transmission of the causative agent^3^.

A better understanding of the pathophysiology of whooping cough is required for the development of new pertussis control strategies. A non-human primate (NHP) model to study whooping cough has become available in recent years^4^. In contrast to other animal models, young baboons (seven to nine months old) display the considerable advantage of developing all clinical symptoms of pertussis disease following *B. pertussis* infection, including paroxysmal cough, nasopharyngeal colonization, and leukocytosis^4^. Moreover, this model has also allowed the study of the effects of pertussis vaccination in NHPs and a comparison of pre-existing commercial and candidate pertussis vaccines^5–8^. Apart from the study of infection in human, which has its limitations, this model appears to be the most appropriate to study bacterial localization and bacteria-host interactions during infection. Previous studies using murine^9^ and swine^10^ models of *B. pertussis* infection provided some evidence of bacterial presence in the nasopharynx and pulmonary bronchi. However, these models do not reproduce the full spectrum of pertussis symptoms and these observations were mainly established after *post-mortem* analysis. It is therefore difficult to make conclusions concerning bacterial colonization and physiologically relevant interactions during the course of the disease. Here, we aimed to evaluate the kinetics of *B. pertussis* colonization and to detect its interactions with antigen-presenting cells (APCs) in the lower respiratory tract of baboons by *in vivo* imaging with probe-based confocal laser endomicroscopy (pCLE) coupled with bronchoscopy. We thus developed and evaluated a juvenile baboon model of infection and transmission with fluorescent *B. pertussis* B1917-GFP, derived from the European clinical isolate B1917^11^ genetically close to D420 US strain previously used in baboon models. Older baboons than the ones used in previously published model, but still juveniles and susceptible to *B. pertussis* infection, were chosen at first to implement this imaging technique as they better support bronchoscopy. This *in vivo* imaging procedure in *B. pertussis*-infected baboons will provide new insights concerning bacterial localization and co-localization with host cells to improve our understanding of pertussis pathogenesis.

## Results

### Generation and characterization of *B. pertussis* B1917-GFP

**Figure 1:**
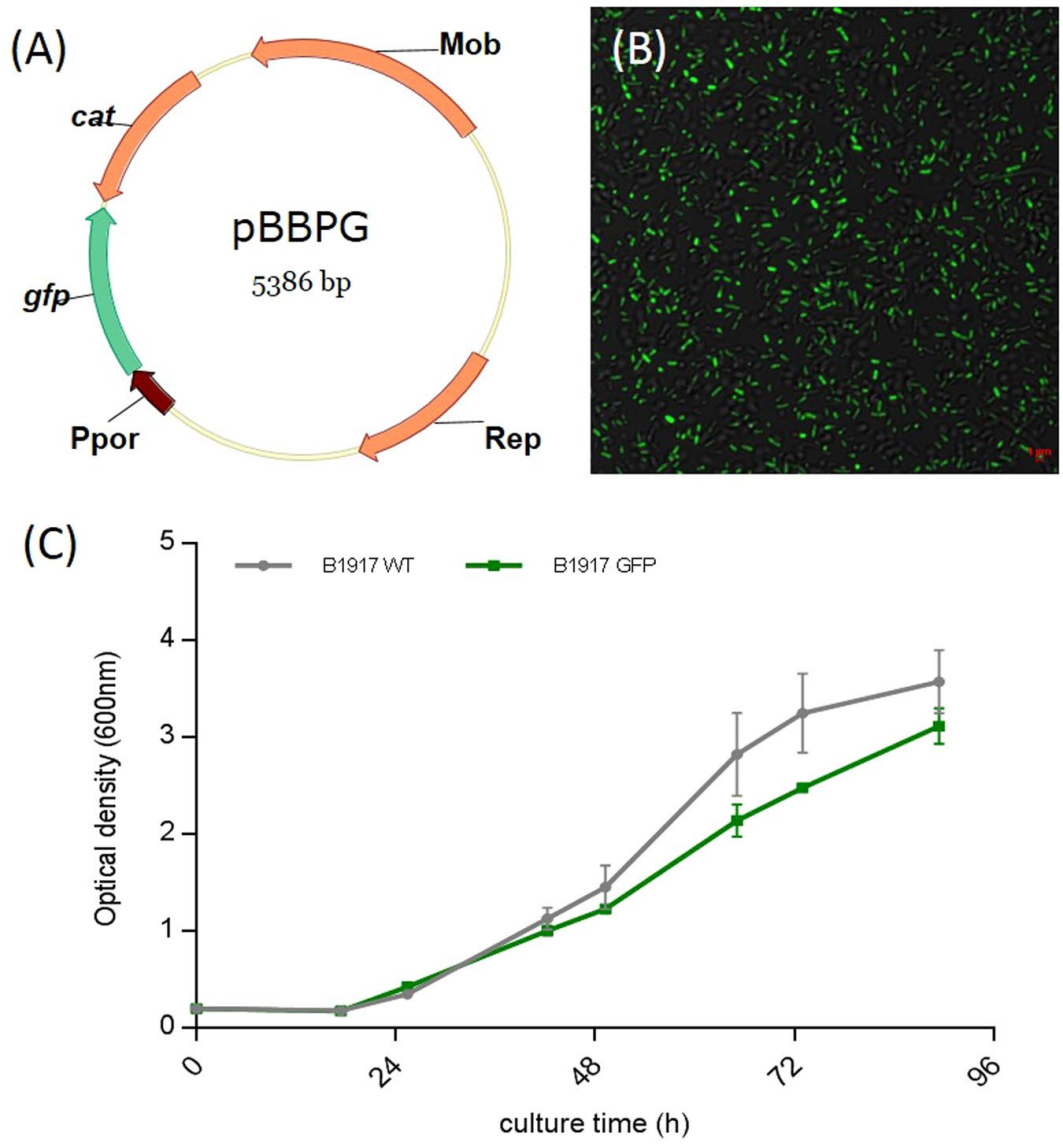
Characteristics of the *B. pertussis* B1917-GFP-expressing strain. (**A**) Plasmid map of the GFP vector, pBBPG, used for transformation. Rep: Plasmid Replication origin, Mob: Mobility gene locus essential for conjugative DNA processing, *cat*: chloramphenicol acetyltransferase (chloramphenicol resistance gene), *gfp*: Green Fluorescent protein gene, Ppor: promotor of the major porin gene from *B. pertussis*. (**B**) GFP bacterial fluorescence observed by confocal microscopy. All bacteria are detected by transmission light (grey) and GFP-expressing bacteria (green) are visible in the focal plane. (**C**) Differential growth of B1917- WT (grey) and B1917-GFP-expressing (green) strains in liquid culture without antibiotics (Stainer-Scholte medium).

The pBBPG vector is a pBBR1MCS^12^ plasmid derivative carrying the GFP-encoded gene under the control of the strong and constitutive *B. pertussis* BPSM porin gene promoter (Ppor) (Fig. 1A). The B1917 fluorescent derivative clone was analyzed by PCR to verify the genuity from the parental European B1917 strain; the sequences showed the fluorescent derivative to contain the *ptxA1*, *prn-2*, and *fim3-2* alleles, like the B1917 and D420 strains.

Fluorescence of the bacteria freshly grown on chloramphenicol-containing BG plates was assessed by confocal microscopy. *B. pertussis* B1917-GFP showed a bright and intense signal after excitation with a 488-nm laser (Fig. 1B).

Previous studies indicated that transformation of *B. pertussis* with a GFP-encoding plasmid did not affect bacterial virulence factor production^13^. We compared the growth of B1917 and B1917-GFP in liquid culture to validate the use of B1917-GFP for *in vivo* infection studies. The growth rate of the GFP-producing bacteria was non-significantly (p = 0.09) lower by 0.22 + /−0.11 OD units during the exponential phase (Fig. 1C).

### Infection of baboons

**Figure 2:**
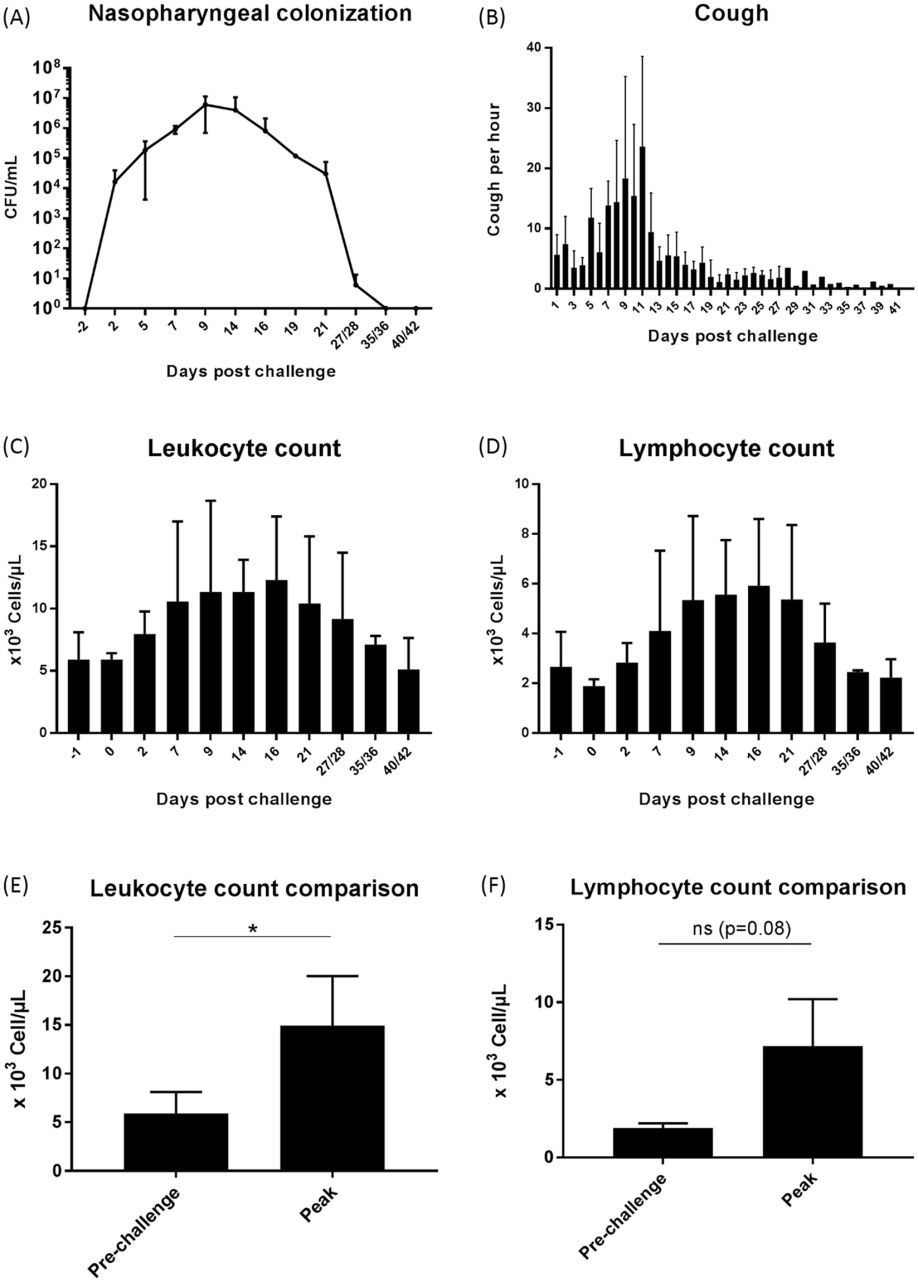
Clinical symptoms of pertussis in baboons exposed to B1917-GFP (n = 3). Nasopharyngeal *B. pertussis* colonization (**A**) was estimated by swab plating on Bordet Gengou blood agar plates. Paroxysmal cough episodes per hour (**B**), circulating white blood cells (**C**) and lymphocytes numbers (**D**) were also analyzed over time. A comparison with paired t-test analysis of leukocytes and lymphocytes counts before challenge and at the peak of infection was also performed (**E**,**F**).

Young baboons have previously been shown to be a relevant model for *B. pertussis* infection and transmission^4,14^. Three animals (39–41 months) were exposed to 10^8^ to 10^10^ CFU of B1917-GFP by intranasal and intra-tracheal routes to investigate the susceptibility of baboons to infection. All animals developed nasopharyngeal colonization and the clinical pertussis symptoms previously described, including leukocytosis and paroxysmal cough (Fig. 2). Nasopharyngeal colonization by the bacteria was detected from day 2 post-inoculation with a maximum of 1.0 × 10^7^ CFU/mL at day 9 (Fig. 2A). We also observed the characteristic paroxysmal cough for all animals with a peak of 23.6 ± 10 coughs per hour during the second week of infection (Fig. 2B). The level of circulating white blood cells also progressively increased (Fig. 2C) with a peak of 14,9 ± 5 × 10^3^ cells/µL (Fig. 2E, p = 0,04) two weeks post-challenge. Among these white blood cells we detected an increase trend of circulating lymphocytes (Fig. 2D,F) but without reaching statistical significance (p = 0,08). Overall, these results show that the B1917-GFP strain can colonize the upper respiratory tract of baboons and induce classical pertussis symptoms during infection. We obtained similar results when we compared exposure of 39-month-old baboons to *B. pertussis* D420 and B1917 (Supplementary Fig. 1).

### *B. pertussis* transmission to naïve co-housed baboons

**Figure 3:**
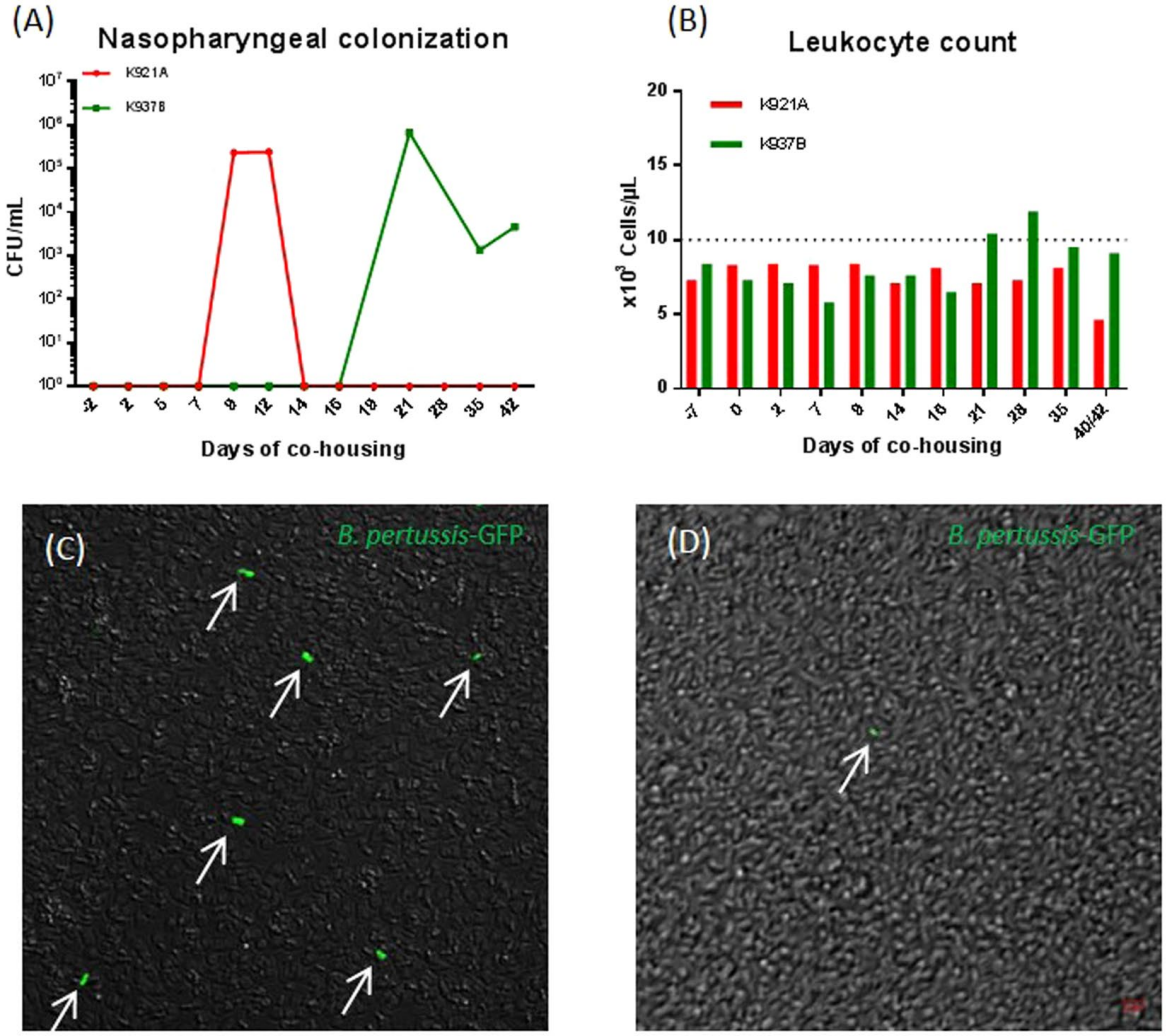
Clinical symptoms of pertussis developed by naive animals co-housed with baboons infected with *B. pertussis*. Nasopharyngeal *B. pertussis* colonization for K921A (red) and K937B (green) (**A**) was estimated by swab plating on Bordet-Gengou blood agar plates. Circulating white blood cell numbers (**B**) were also measured over time. The dotted line corresponds to estimated pathological WBC counts. Fluorescence of recovered nasopharyngeal *B. pertussis* was assessed by confocal fluorescence imaging as soon as bacteria were first detected in swabs at D9 for K921A (**C**) and D20 for K937B (**D**). All bacteria were detected in grey by transmitted light microscopy and GFP expressing *B. pertussis* were detected in green (arrows) by confocal fluorescence microscopy. Scale bar: 2 μm.

Airborne and direct transmission of *B. pertussis* between baboons has been previously demonstrated for young animals^14^. Here we performed two duplicate experiments to assess direct B1917-GFP transmission between juvenile animals. In each experiment, a 39-month-old naïve baboon (identified as K921A and K937B) was co-housed in the same cage unit with another animal of the same age challenged with *B. pertussis* B1917-GFP. Both co-housed naïve animals were colonized by *B. pertussis* in the nasopharynx with diverse kinetics. One animal, K921A, was briefly colonized for one week after 9 days of co-housing with the infected animal (Fig. 3A). The second baboon, K937B, was colonized from D21 post co-housing, but the bacteria were recovered from the nasopharynx for more than three weeks (Fig. 3A). Hence, some fluorescent bacteria were detected in the suspensions of nasopharyngeal swabs at D9 for K921A and D20 for K937B by confocal microscopy, confirming colonization of both baboons by the B1917-GFP strain carried by the co-housed infected baboon (Fig. 3C,D). Only K937B showed an increase of circulating WBCs from the beginning of nasopharyngeal colonization (Fig. 3B). These data indicate that juvenile baboons can transmit B1917-GFP from one animal to another.

### *Ex vivo* imaging of baboon airways using pCLE

**Figure 4:**
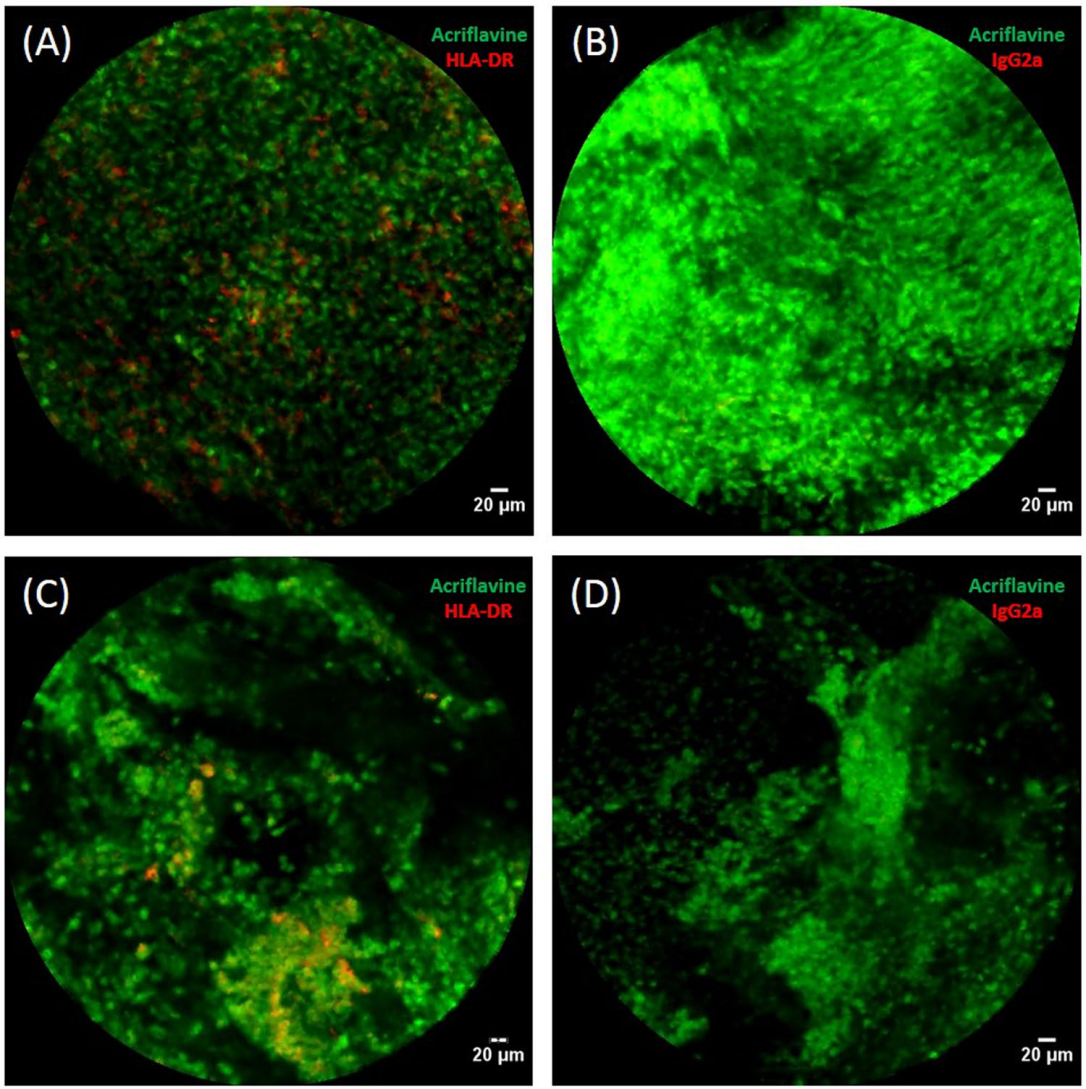
Imaging of baboon airways by probe-based confocal laser endomicroscopy (pCLE). *Ex vivo* pCLE images of tracheal (**A**,**B**) and lung (**C**,**D**) explants. Prior to *ex vivo* imaging, tissues were non-specifically stained with acriflavine (green) and with either anti-human HLA-DR AF647 (**A**,**C**) or isotype control IgG2a AF647 antibodies (**B**,**D**) (red).

*Ex vivo* imaging was performed using baboon tissues to establish pCLE technique in airways. APC detection was assessed using this technique as they have been shown to play a key role in the early immune response against *B. pertussis*^15–18^. APCs were selectively labeled and detected in the lower respiratory tract, from the trachea (Fig. 4A,B) to the alveoli (Fig. 4C,D), with anti-HLA-DR-AF647 (Fig. 4A,C), whereas there was no detectable signal with the IgG2a-AF647 control (Fig. 4B,D). APC labeling specificity was confirmed by confocal visualization of frozen tissue slices (Supplementary Fig. 2). Overall, these results show that APCs can be selectively labeled and tracked in the lower respiratory tract of baboons by pCLE imaging.

### *In vivo* imaging of *B. pertussis* colonization in infected baboons

**Figure 5:**
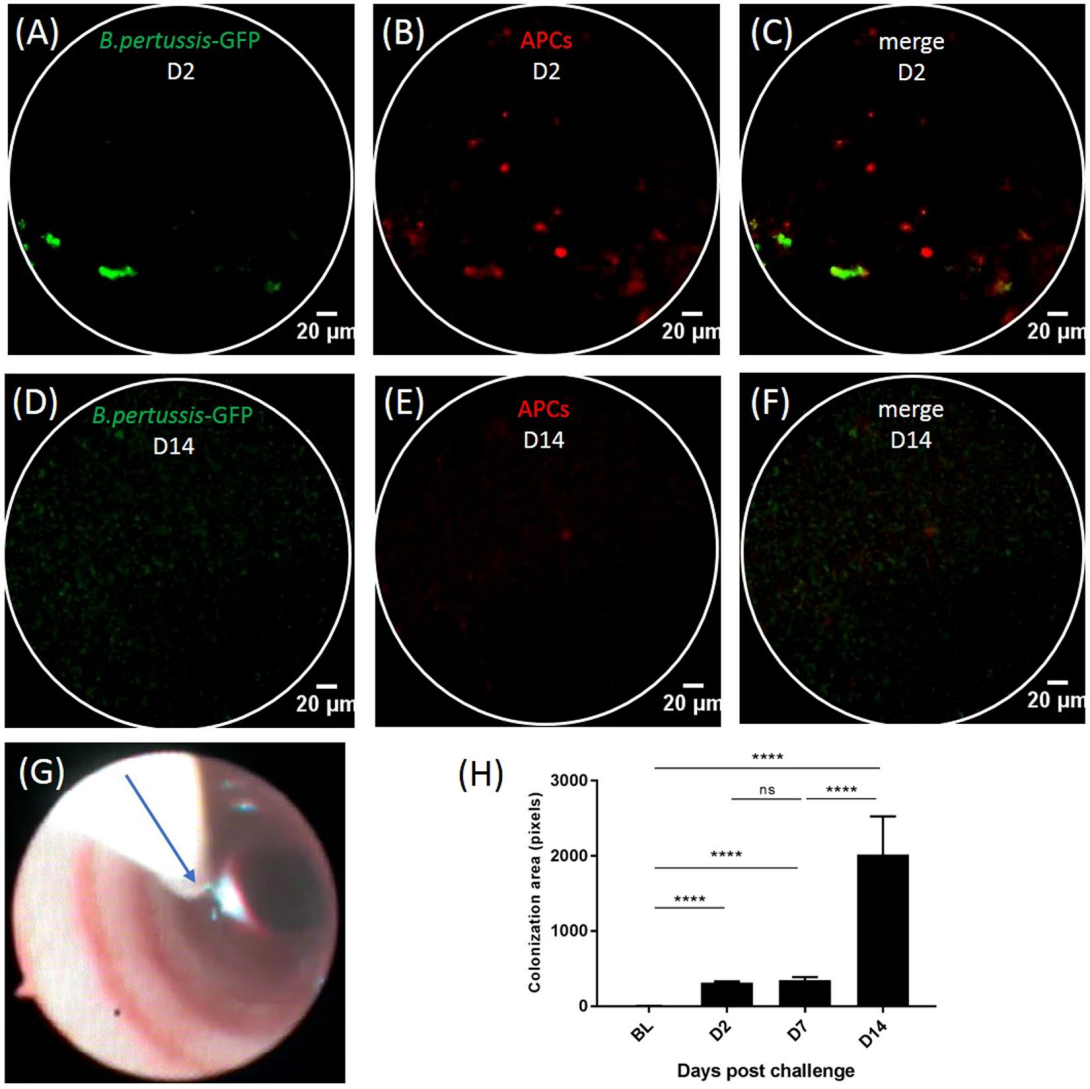
*In vivo* imaging by pCLE coupled with bronchoscopy in *B. pertussis* B1917-GFP infected baboons. Images (**A**–**F**) extracted from pCLE acquisition in the trachea at D2 (**A**–**C**) and D14 (**D**–**F**) post-infection of one animal, representative of the three challenged baboons. B1917-GFP bacteria (aggregates and biofilms) are detected in 488-nm channel (green) (**A**,**D**) and APCs are labeled with anti-HLA-DR AF647 antibody and detected in the 660-nm channel (red) (**B**,**E**). (**G**) Image taken with the bronchoscope camera showing the pCLE probe (arrow) in the trachea. (**H**) Evaluation of *B. pertussis* B1917-GFP colonization in the trachea determined by the area of GFP signal in the 488-nm channel (n = 3 animals) and comparison of the data between timepoints (****p < 0.0001).

We reproducibly performed *in vivo* bronchoscopy on 39-month-old baboons down to the secondary bronchi without affecting oximetry or cardiac rate. *In vivo* bronchoscopy coupled with pCLE imaging was performed on naïve and *B. pertussis* B1917-GFP-infected baboons (Fig. 5A–G). Previously published data on human airway pCLE imaging reported a significant signal in the green channel due to endogenous auto-fluorescence of elastin fibers, located behind the epithelial barrier^19^. Surprisingly, there was almost no auto-fluorescence in the green channel at the tracheal and bronchial surface in the naïve animals prior to challenge (Fig. 5H).

**Figure 6:**
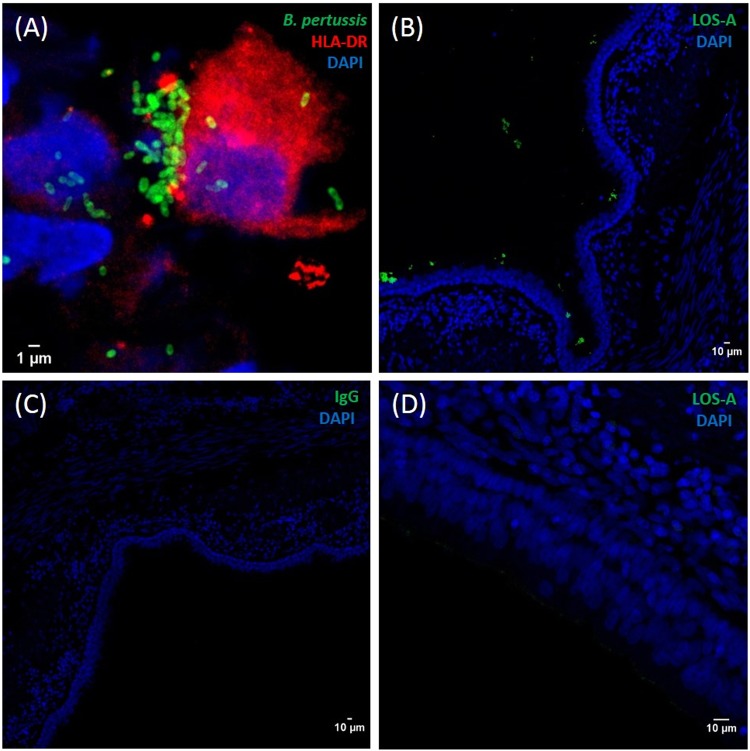
Immunohistofluorescence stainings. (**A**) APC staining with HLA-DR antibodies (red) after *ex vivo* baboon lung co-culture with *B. pertussis* B1917-GFP (green). (**B**–**D**) Post-mortem (five weeks post-inoculation) tissue analysis on *B. pertussis* challenged (**B**,**C**) or naive (**D**) animals. Immunohistofluorescence staining of frozen bronchial slices with either polyclonal anti-*B. pertussis* LOS-A mouse antibodies (**B**,**D**) or nonspecific mouse IgG3 (**C**) (green). Nuclei are stained with DAPI (blue).

We detected co-localized GFP and HLA-DR-AF647 signals for all infected animals at D2 post-challenge (representative images for one animal in Fig. 5A–F), confirming the *ex vivo* co-culture findings (Fig. 6A). The GFP signal was detectable in the trachea of all *B. pertussis* B1917-GFP-infected animals from D2 (Fig. 5A,C) and increased up to D14 (Fig. 5D,F), indicating progressive bacterial colonization of the lower respiratory tract during the early phase of infection (Fig. 5H). However, there was no GFP signal after D21 in challenged or non-challenged co-housed animals, despite *B. pertussis*-positive nasopharyngeal cultures (Fig. 3), most likely due to the progressive loss of the bacterial fluorescence over time (Supplementary Fig. 3).

We also detected *B. pertussis* bacteria *post mortem* by IHF in the trachea and bronchi of infected baboons five weeks post-inoculation (Fig. 6B), whereas no signal was observed with the isotype control (Fig. 6C) nor in naïve animals (Fig. 6D).

## Discussion

Recent pertussis outbreaks in many countries with high vaccination coverage have highlighted the crucial need to improve current vaccination strategies. This may be aided by better knowledge concerning the pathogenicity of pertussis disease. *In vitro* and preclinical animal studies, mostly in mice, have revealed diverse aspects of pertussis pathogenicity, immunomodulatory effects of bacterial virulence factors and protective immune responses to *B. pertussis* infection^16–18,20–23^. However, these models do not reproduce the full spectrum of pertussis symptoms, most notably the cough. The recently developed baboon model of whooping cough has made it possible to study the modalities of pertussis transmission, providing strong support for the asymptomatic carriage hypothesis, as well as evaluating the efficiency of pertussis vaccines. A better understanding of how *B. pertussis* interacts with this host and a comparison with other models or *in vitro* data will help to build a strong basis for future vaccine development.

Here, we developed and applied *in vivo* imaging technologies to study bacterial localization and to evaluate bacterial-host cell interactions in *B. pertussis-*infected baboons. We have successfully constructed a GFP-producing *B. pertussis* strain from clinical isolate B1917, belonging to the *ptxP3*-*ptxA1* clade, as a representative of currently circulating strains of *B. pertussis*^11^. Infection of juvenile baboons with this fluorescent strain induced classical pertussis symptoms. The kinetics of bacterial colonization in the upper airways was comparable to previous published data for younger baboons infected with the *B*. pertussis D420 strain^24^. We also observed characteristic paroxysmal cough, especially in the first two weeks of infection, but with residual cough for up to one month. The intensities of the general symptoms appear to be milder in B1917-GFP-infected 39-month-old baboons than in young juveniles challenged with *B. pertussis* D420^4–6^. This observation is consistent with the reduced and more variable clinical illness observed in adult humans relative to infants^25^. An investigation of the influence of strain or GFP synthesis on clinical pertussis symptoms in comparison with the infection profile of 39-month-old baboons revealed no major differences in the clinical symptoms induced by inoculated with B1917-GFP, B1917 or D420 strains, indicating that GFP production did not significantly affect clinical symptoms. We used the GFP derivative strain to investigate natural *B. pertussis* transmission to naïve co-housed baboons and confirmed previous published data from younger animals^14^. However, the efficiency of transmission to naïve baboons and the intensity of associated clinical symptoms may vary due to social interactions and inter-individual variability. Nevertheless, effective clinical infection of the challenged animals validated B1917-GFP as an appropriate strain for the study of pertussis pathogenesis by imaging. This allowed us to investigate bacterial-host cell interactions using *ex vivo* and *in vivo* imaging technologies.

We used this baboon infection model to investigate whether cell populations of interest, such as APCs, and bacteria could be labeled and detected *in vivo* during a *B. pertussis* infection by pCLE imaging. The imaging technologies used here are well described for the study of human airways, but have not yet been extensively used in preclinical models without invasive surgery or euthanasia^26^. Unexpectedly, only low auto-fluorescence was observed in baboons relative to human airways, where elastin fibers induce strong background fluorescence. This difference may be explained by species specificities^26^ or the young age of the animals used here. We were therefore able to specifically detect progressive bacterial colonization of the trachea in infected animals during the first two weeks of infection by pCLE, as well as co-localization of the pathogen with APCs. This provides *in vivo* confirmation of the strong interactions previously described between *B. pertussis* and macrophages and dendritic cells of the airways^27^ but so far, precise bacterial localization, extra or intra-cellular, still need to be confirmed. APC labeling by topical application of fluorescent anti-HLA-DR antibodies has been successfully implemented in the tracheal and bronchial mucosa in baboons, but had to be repeated every week for optimal detection, as described for a skin model^28^. This is the first description of non-invasive *in vivo* bacterial detection in airways by optical imaging in NHP. A study in rats has described lung colonization by *Aspergillus fumigatus* using external detection by pCLE, but this required invasive surgery and was performed only at day 5 post-challenge^26,29^. The spatial resolution of pCLE probes used in this study, with a lateral resolution of 3.5 μm, prevented us from detecting single bacteria, but we could detect aggregates or biofilms as already demonstrated by confocal microscopy in mice^9^. Another limitation to bacterial infection imaging in our study was the gradual loss of plasmid-mediated fluorescence in the absence of selective pressure. This led to the progressive loss of fluorescence *in vivo*, which prevented us from following bacterial colonization by imaging for more than two weeks. However, long-term *B. pertussis* colonization may be studied in baboons using our imaging technologies with a *B. pertussis* strain that strongly and stably expresses a chromosomally inserted *gfp* gene. This pathophysiological imaging study in juvenile baboons demonstrates that it is possible to follow bacterial colonization of the lower respiratory tract and explore host-pathogen interactions during infection with minimal invasiveness. Our results open many new opportunities to characterize colonization by *B. pertussis* and the cellular populations involved in the local *in vivo* response to *B. pertussis* infection in detail. This should lead to an improved understanding of the pathogenesis of whooping cough in primates.

## Materials and Methods

### Ethics statement

All *in vivo* studies were performed on juvenile *Papio Anubis* (39–41 months). Baboons were obtained from the *Station de Primatologie* (CNRS, Rousset-sur-Arc, France) and housed in the facilities of the Infectious Disease Models and Innovative Therapies (IDMIT) center at the “Commissariat à l’Energie Atomique et aux Energies Alternatives” (CEA, Fontenay-aux-Roses, France). The animals were used in accordance with French national regulations (CEA Authorization Number A92-032-02). The CEA complies with the Standards for Humane Care and Use of Laboratory Animals of the Office for Laboratory Animal Welfare (OLAW, USA) under OLAW Assurance number #A5826-01. The use of NHPs at the CEA complies with the recommendations of European Directive 2010/63 (recommendation N°9). These experiments were approved by the ethics committee number 44 of the French Ministry of Research (statement number A16-041). Experimental procedures (animal handling, bacterial inoculation, and blood sampling) were conducted after animal sedation with ketamine chlorydrate (Rhône-Mérieux, France, 10 mg/kg) or Zoletil®100 (Virbac, France, 5 mg/kg). Tissues were collected at necropsy: animals were sedated with ketamine and then euthanized by intravenous injection of 180 mg/kg sodium pentobarbital.

### Bacterial strains and growth media

*B. pertussis* clinical isolates B1917 and D420^24^ were provided by Frits Mooi and came from the RIVM collection (Bilthoven, The Netherlands). The B1917 strain was transformed with pBBPG^30^, encoding green fluorescent protein (GFP) (Fig. 1A). Bacterial fluorescence was verified on slices using the Nikon-A1R confocal laser-scanning microscope attached to an inverted ECLIPSE-Ti (Nikon, Japan).

*B. pertussis* strains were grown in Bordet-Gengou agar plates containing 10% (v/v) sheep blood (BG plates, BD) for 72 h at 37 °C. For growth studies, bacteria were suspended in modified Stainer-Scholte medium supplemented with cyclodextrin (1 g/L) and casamino acids (5 g/L) without any antibiotics^31^ at an OD_600nm_ of 0.15. Liquid cultures were incubated at 37 °C and 320 rpm in disposable 15-mL flasks. Optical density OD_600nm_ was measured twice a day to evaluate bacterial growth. Cultures were performed in four replicates.

### Preparation of inoculum and infection of baboons

*B. pertussis* colonies grown on BG plates for 72 h at 37 °C were resuspended in sterile phosphate-buffered saline (PBS) at an OD_600nm_ of 0.9, which corresponds to a density of 10^8^ to 10^9^ CFU/mL. Baboons were infected by intranasal and intra-tracheal routes as previously described^4,32^. The inoculum concentration was validated by serial dilutions and plating on BG plates and colony counting after seven days of incubation at 37 °C. Automatic colony counting was performed using a Scan®300 colony counter (Interscience, France).

### Bacterial colonization and monitoring of infection

Bacterial content in the nasopharynx was measured twice weekly using flocked swabs in each naris of animals. Samples were then collected in liquid Amies medium (COPAN, USA). This solution (50 μL) was plated in duplicate at various dilutions on BG plates. The number of colony-forming units (CFU) per plate was estimated by automatic counting after seven days of incubation. Whole blood was evaluated for the number of circulating white blood cells and lymphocytes by a complete blood count. Baboons were euthanized six weeks after challenge.

### Quantification of coughing

Animal cages were monitored with a video-recording system. Cough occurrences for each animal were assessed daily during a 6-h period (6:00–9:00 a.m.; 5:00–8:00 p.m.) using Windows Movie Maker software.

### Antibody labeling

Mouse anti-human HLA-DR antibody that targets APCs (BD, clone L243) and its isotype control (mouse IgG2a (BD)) were labeled with AlexaFluor647 using the Microscale Protein Labeling Kit (Invitrogen). Antibody and fluorophore concentrations were measured after coupling by nanodrop spectrophotometry.

### *Ex vivo* imaging of the baboon respiratory tract

Lower respiratory tract explants were obtained from euthanized baboons. Immediately after euthanasia, lung lobes were isolated and tracheal rings cut and preserved in complete medium (DMEM containing 10% SVF, NEAA (1X concentration), 1 mM sodium pyruvate, and 0.01 M HEPES) at 37 °C. A solution of anti-HLA-DR or IgG2a mouse antibody labeled with AlexaFluor647 (200 µL, 100 µg/mL) was dropped into the bronchus of a lobe and the sample incubated for 2 h at 37 °C. Tracheal rings were submerged in the same antibody solution (200 µL, 100 µg/mL) for 2 h at 37 °C. Then, a solution of acriflavine (Sigma Aldrich; 0.05 mg/mL; 1 mL) was topically administrated to the main bronchus of each lobe and tracheal rings submerged in the same solution for 5 min before imaging. Such acriflavine RNA staining allows detection of all cellular structures at 488 nm. No rinsing was performed to avoid modification of the alveolar structure and cell composition due to the lavage. Probe-based Confocal Laser Endomicroscopy (pCLE) was performed using the Cellvizio DualBand® device (MaunaKea Technologies, France) with S-1500 or miniZ probes. For tracheal ring imaging, probes were deposited directly on the tracheal epithelial surface. For lung lobe imaging, probes were inserted into the main bronchus and directed down to the alveolar structures. For *ex vivo* co-culture experiments with B1917-GFP, acriflavine was replaced by the bacterial inoculum (1 mL, 10^8^ CFU) and samples were incubated 48 h at 37 °C.

### *In vivo* pCLE imaging of the baboon respiratory tract

Xylocaine (5%; Aspen Pharma, France) was topically sprayed into the back of the mouth of anaesthetized baboons. Bronchoscopy was performed using a Karl Storz Special fibroscope. Anti-HLA-DR-AF647 antibodies (200 µL, 100 µg/mL) were first topically dropped at the top of the trachea and into the left main bronchus via the working channel of the endoscope to label the APCs. After 2 h of incubation, pCLE with bronchoscopy was performed as previously described^19^. Briefly, the optical miniZ probe was inserted into the working channel of the bronchoscope and imaging sequences performed from the top of the trachea down to the secondary bronchi, especially in the area where the antibodies were dropped. APCs were detected at 660 nm and *B. pertussis* B1917-GFP at 488 nm. Signal background in these two channels was assessed before baboon challenge, in the absence of fluorescent bacteria and antibodies. *In vivo* imaging sequences were performed once a week for four weeks starting seven days prior to animal infection with *B. pertussis*.

### Immunohistofluorescence

Tissues (lungs, bronchi, and trachea) were first fixed with 4% PFA for 24 h at 4 °C. Samples were then dehydrated in 30% sucrose for 24 h, embedded in OCT, and finally frozen in isopentane and liquid nitrogen. *B. pertussis* was detected in 10-µm tissue sections using anti-*B. pertussis*-LOS-A (ThermoFisher, clone D26E) and goat anti-mouse IgG3-AlexaFluor594 (Life Technologies) antibodies. APCs were detected using anti-human-HLA-DR and goat anti-mouse IgG2a-AlexaFluor488 (Life Technologies) antibodies. Cell nuclei were stained with 4′,6-diamidino-2-phenylindole (DAPI). Image acquisition was performed using a SP8 confocal microscope (Leica, Germany).

### Image analysis

Films obtained using pCLE were first selected using IC-Viewer software and then exported to ImageJ (National Institute of Mental Health, USA). Intensity thresholds were fixed to minimize the background to signal ratio. Bacterial colonization of the lower airways was assessed using GFP area quantification. Briefly, the total area of GFP-positive signal was calculated for each imaging time-point using Visiopharm® Software (Visiopharm, Denmark) in 30 images randomly extracted from the pCLE acquisition. No signal size restrictions were applied due to the small size of the bacteria. For *post-mortem* tissue analysis, acquisitions performed on the SP8 confocal microscope were analyzed using ImageJ.

### Statistical analysis

Data are reported as the means ± standard errors for replicate experiments. For bacterial growth experiments and colonization assessment by pCLE, unpaired t-tests were conducted to detect statistical differences (p < 0.05). Paired t-tests were also conducted to compare levels of leukocyte and lymphocyte counts before challenge and at the peak of infection (GraphPad Prism software).

## Electronic supplementary material

supplementary dataset

## Data Availability

All data generated or analyzed during this study are available from the corresponding author on reasonable request.
