# Supplementary material for: In vivo imaging of bacterial colonization of the lower respiratory tract in a baboon model of Bordetella pertussis infection and transmission
Source: Sci Rep. 2018 Aug 16;8:12297. doi: 10.1038/s41598-018-30896-7 (PMC6095854; doi:10.1038/s41598-018-30896-7)
Supplement: Supplementary file 1 — supplementary dataset [file 41598_2018_30896_MOESM1_ESM.pdf]

In vivo imaging of bacterial colonization of the lower respiratory tract in a baboon model of *Bordetella pertussis* infection and transmission

Thibaut Naninck[a], Loïc Coutte[b], Céline Mayet[a], Vanessa Contreras[a], Camille Locht[b], Roger Le Grand[a], and Catherine Chapon[a]

(a) CEA – Université Paris Sud 11 – INSERM U1184, Immunology of Viral Infections and Autoimmune Diseases, IDMIT Department, IBFJ, Fontenay-aux-Roses & Le Kremlin-Bicêtre, France

(b) Center for Infection and Immunity of Lille - INSERM U1019- CNRS UMR8204, Institut Pasteur de Lille, Lille, France

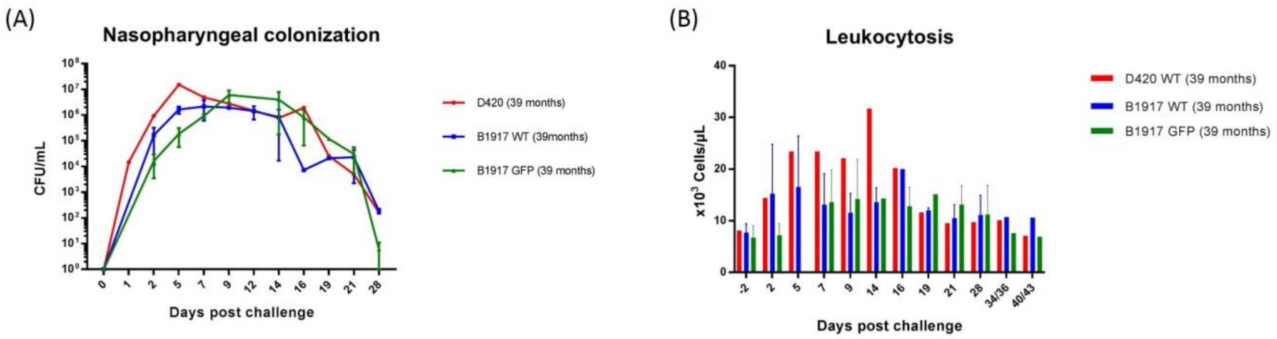

Supplementary figure 1. Clinical symptoms developed by baboons challenged with *B. pertussis* strains D420 wild-type (red, n=1), B1917 wild-type (blue, n=2) or B1917-GFP (green, n=2). Nasopharyngeal *B. pertussis* colonization (A) was estimated by swab plating on Bordet Gengou blood agar plates. Circulating white blood cell numbers (B) were also measured over time.

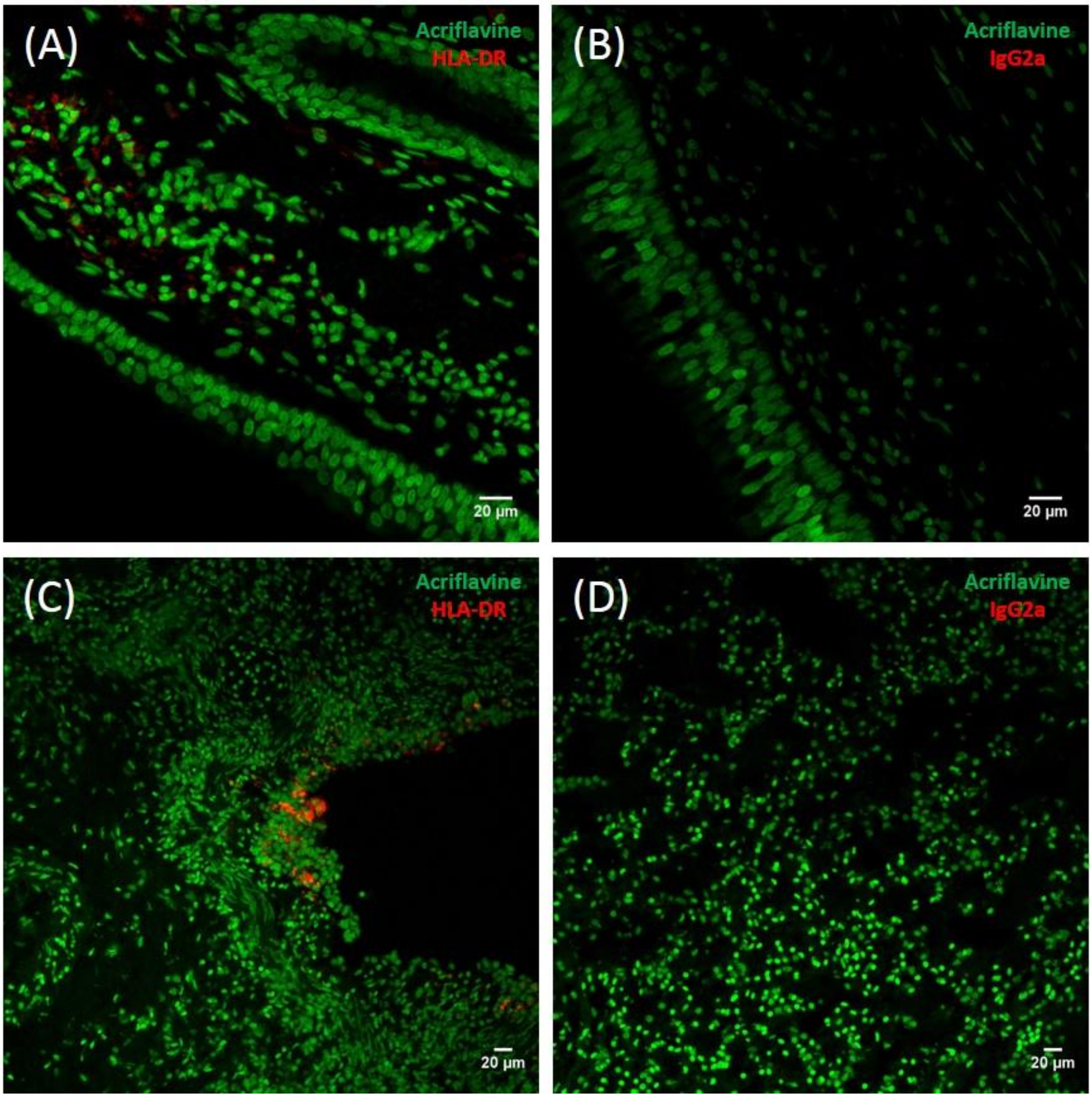

Supplementary figure 2. Prior to *ex vivo* pCLE imaging, baboon tissues were nonspecifically stained with acriflavine (green) and either anti-human HLA-DR AF647 (A, C) or isotypic control IgG2a AF647 antibodies (B, D) (red). Tracheal (A-B) and lung (C-D) frozen tissue sections were then observed with a confocal microscope.

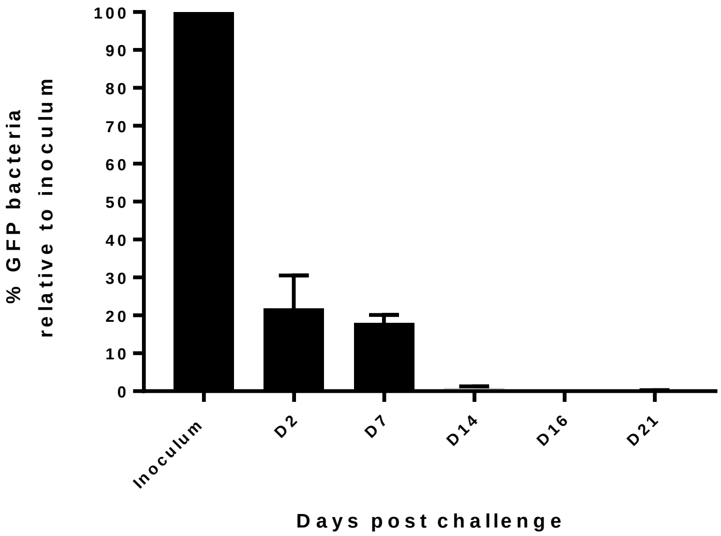

Supplementary figure 3. Percentage of nasopharyngeal GFP-positive *B. pertussis* relative to inoculum over time in nasopharyngeal swab samples isolated from one infected baboon. Fluorescence was assessed in 10 pictures by confocal microscopy after seven days of nasopharyngeal swab culture on non-selective Bordet-Gengou agar plates and DAPI staining.
